# Biopsied non-dental plaque-induced gingival diseases in a Chinese population: a single-institute retrospective study

**DOI:** 10.1186/s12903-021-01614-z

**Published:** 2021-05-17

**Authors:** Xiaotian Li, Jianyun Zhang, Heyu Zhang, Tiejun Li

**Affiliations:** 1 Department of Oral Pathology, National Clinical Research Center for Oral Diseases, Peking University School and Hospital of Stomatology, Beijing, China; 2 Central Laboratory, Peking University School and Hospital of Stomatology, Beijing, China; 3 Research Unit of Precision Pathologic Diagnosis in Tumors of the Oral and Maxillofacial Regions, Chinese Academy of Medical Sciences (2019RU034), Beijing, China

**Keywords:** Non-dental plaque-induced gingival diseases, Classification of gingival health and gingival disease/conditions, Epidemiology

## Abstract

**Background:**

While inflammatory diseases such as gingivitis and periodontitis induced by dental plaque biofilms constitute the majority of gingival lesions, gingiva can also be affected by a variety of diseases with aetiologies different from bacterial biofilms. The aim of this study was to retrospectively analyze the frequency and distribution of non-dental plaque-induced gingival diseases (NDPIGDs) in the Chinese population in a single institute.

**Methods:**

A total of 6859 samples of biopsied gingival diseases during the period 2000–2019 were obtained from the Department of Pathology, Peking University Hospital of Stomatology. Lesions were categorized by histopathological diagnosis, pathological characteristics and the new classification of gingival health and gingival diseases/conditions. Demographic information, such as gender, location, and age, were also analyzed.

**Results:**

Among 6859 biopsied NDPIGD samples, the five most frequent diagnoses included oral squamous cell carcinoma (OSCC, n = 2094), fibrous hyperplasia (n = 2026), pyogenic granuloma (n = 478), epithelial dysplasia (n = 477), and epithelial hyperplasia/hyperkeratosis (n = 436). All types could be grouped into nine categories according to their pathological characteristics. The most common biopsied NDPIGDs category was “hyperplastic lesions” (n = 2648, 38.61%), followed by “malignant neoplasms” (n = 2275, 33.17%). The most common diagnosis types in each category were fibrous hyperplasia and OSCC. Of all NDPIGDs, most lesions could be categorized into the new classification of gingival health and gingival diseases/conditions; only 7.07% did not fit the current classification system.

**Conclusions:**

The present study is the first report on the frequency and distribution of biopsied NDPIGDs in a Chinese population. Unlike previous studies, the most prevalent categories were “hyperplastic lesions” and “malignant neoplasms”. The proportion of “malignant neoplasms” and “oral potentially malignant disorders” was remarkably higher than in previous researches. Nevertheless, the study provided epidemiological information on many NDPIGDs, which could be useful for future health policies as well as screening programs.

## Background

As one of the most common sites for various pathogens affecting oral health, gingiva has long been the concentration scope of many clinicians. While most lesions that occurr on oral gums are inflammatory diseases induced by dental plaque biofilm [1]. gingiva can also be affected by a variety of neoplastic or non-neoplastic conditions that show aetiologies different from bacterial biofilm [2]. Accurate diagnosis is essential for better management of these lesions because of their various effects on clinical behavior and the required treatment.

Non-dental plaque-induced gingival diseases (NDPIGDs) are defined as a group of lesions that are not caused by bacterial plaque and often do not disappear after dental plaque removal; however, it is emphasized that the severity of clinical manifestations sometimes relies on the interaction with the remaining bacterial plaque [3, 4]. These lesions were not identified until the classification of periodontal diseases and conditions drawn up by the American Academy of Periodontology (AAP) in 1999. The system named and classified a wide range of diseases including gingival diseases induced by specific bacteria, viruses and fungi, genetic gingival lesions, gingival manifestations of systemic conditions, traumatic lesions, foreign body reaction, and conditions not otherwise specified. However, as mentioned above, the gingiva can be affected by many types of non-neoplasms or neoplasms (either benign or malignant), which were not included in the 1999 classification system. A new classification scheme for periodontal and peri-implant diseases and conditions was established by the European Federation of Periodontology and AAP in 2018 [5]. In this classification, some modifications were made, and neoplasms such as leukoplakia and squamous cell carcinoma were also included. From a pathological perspective, their aetiologies might be different; however, periodontists may be unaware of them due to the limited number of reported studies. Therefore, knowledge of the prevalence and distribution of these lesions could offer help in regional screening programs concerning oral health [6].

To date, limited studies focused on gingival lesions have been reported in different countries [7–11]. Pyogenic granuloma and fibroma constitute the majority of non-neoplastic lesions that are most likely seen in the gingiva [7–9, 12]. Fibroma is the most common type among the neoplastic diseases while the proportion of oral squamous cell carcinoma (OSCC) accounts for majority of the malignancies [8, 12]. Drug-induced gingival enlargement is a common adverse effect in patients treated with anticonvulsant, immnosuppressant and antihypertensive medications. An increasing number of reports were associated with drug-induced gingival enlargement, investigating the effects of different kinds of drugs as well as their possible molecular mechanisms behind [13–15]. Besides, gingival lesions are characteristic for many rare diseases and could be one of the early manifestations before multiple organs’ involvement [16, 17]. It is reported that many rare diseases with an orofacial involvement showed periodontally manifestations, indicating the importance of early diagnoses of these underlying diseases. However, among all the studies with valid references, none provide a summary concerning the epidemiology of NDPIGDs in a Chinese population. In addition, most of these investigations simply recorded the epidemiology of NDPIGDs despite the periodontal classification of these diseases. While one study combined the histopathological diagnosis with periodontal classification, it was mostly based on the classification of 1999 [11]. With the presence of a new classification of periodontal lesions proposed in 2018, it is essential to be aware of the epidemiology of NDPIGDs. Therefore, the aim of this study was to retrospectively analyze the frequency and distribution of NDPIGDs in the Chinese population in a single institute.

## Methods

This research was approved by institutional review board of Peking University Hospital of Stomatology (Approval No. PKUSSIRB-201418116a), and written informed consent was obtained from all patients. A retrospectively study was performed on gingival biopsies obtained during a 20-year period from 1 January 2000 to 31 December 2019, in the Department of Oral Pathology, Peking University Hospital of Stomatology, and all processes were in accordance with the ethical standards laid down in the 2008 Declaration of Helsinki and its later amendments. We reviewed the medical records of all patients those had undergone biopsies during this period. In routine procedures, tissues captured by clinical doctors were fixed in formalin and then transferred to the Department of Pathology. Tissues were made into FFPE samples and slides were used for histopathological diagnosis, which was made by 2–3 independent pathologist. Histopathological examination was the only method of diagnosis in all samples. The inclusion criteria were as follows: (a) diseases affected the gingiva with adequate and definite histopathological diagnosis; (b) cases with adequate demographic information. Exclusion criteria included: salivary gland diseases and intraosseous lesions that extended to the gingiva; gingivitis or nonspecific inflammatory diseases that could not be distinguished from gingivitis or periodontitis. A total of 8456 cases were collected during the period of 2000–2019 and 6859 cases left after exclusion for final analysis. Data regarding age, sex, type, location, and histopathological diagnoses of the lesions were collected from each subject. Data entry and analysis were performed by using Microsoft Excel software and GraphPad Prism9. The Chi-squared (χ^2^) test was used to analyze the gender, location and age distribution of the lesions.

## Results

A total of 8456 cases were collected during the period of 2000–2019. A number of 1032 samples were salivary gland diseases and intraosseous lesions. Besides, 389 cases were excluded for unknown demographic information and 176 samples were also excluded due to ambiguous diagnosis. Therefore, 6859 cases left for further analysis. The number and frequency of the biopsied samples are summarized in Table 1. Among a total of 6859 biopsied NDPIGD samples, the five most frequent types of all cases included OSCC (n = 2094, 30.53%), fibrous hyperplasia (n = 2026, 29.54%), pyogenic granuloma (n = 478, 6.97%), epithelial dysplasia (n = 477, 6.95%) and epithelial hyperplasia/hyperkeratosis (n = 436, 6.36%). Specifically, OSCC ranked at the top of all NDPIGDs, indicating that OSCC was the most severe lesion encountered in the gingiva. The sixth most frequent diagnosis as well as the most common type of autoimmune disorder was pemphigoid (n = 298), which accounted for 4.34% of all lesions. Lesions with more than 50 cases also included lichen planus (n = 290, 4.23%), pemphigus (n = 86, 1.25%), malignant melanoma (n = 86, 1.25%), verruciform xanthoma (n = 84, 1.22%), papilloma (n = 79, 1.15%) and melanotic macules (n = 51, 0.74%).

**Table 1:** The number and frequency of different biopsied NDPIGDs according to diagnosis

Type	Total no. (%)
Oral squamous cell carcinoma	2094 (30.53)
Fibrous hyperplasia	2026 (29.54)
Pyogenic granuloma	478 (6.97)
Epithelial dysplasia	477 (6.95)
Epithelia hyperplasia/Hyperkeratosis	436 (6.36)
Pemphigoid	298 (4.34)
Lichen planus	290 (4.23)
Pemphigus	86 (1.25)
Malignant melanoma	86 (1.25)
Verruciform xanthoma	84 (1.22)
Papilloma	79 (1.15)
Melanotic macules	51 (0.74)
Hereditary gingival fibromatosis	40 (0.58)
Peripheral ameloblastoma	40 (0.58)
Malignant lymphoma	33 (0.48)
Peripheral giant cell granuloma	32 (0.47)
Drug-induced gingival enlargements	28 (0.41)
Peripheral odontogenic fibroma	28 (0.41)
Exogenous pigmentation	28 (0.41)
Soft tissue sarcoma	24 (0.35)
Spindle cell sarcoma	22 (0.32)
Nevus	21 (0.31)
Vascular malformations	18 (0.26)
Lichenoid lesion	11 (0.16)
Metastatic carcinoma	6 (0.09)
Myofibroma	5 (0.07)
Hemangioma	5 (0.07)
Malignant peripheral neurolemmoma	4 (0.06)
Neurofibromatosis	4 (0.06)
Neurolemmoma	3 (0.04)
Inflammatory myofibroblastic tumor	3 (0.04)
Leukemia	2 (0.03)
Myoepithelial carcinoma	2 (0.03)
Fibrous histiocytoma	2 (0.03)
Plasma cell gingivitis	2 (0.03)
Tuberculosis	2 (0.03)
Lipoma	2 (0.03)
Solitary fibrous tumor	2 (0.03)
Congenital epulis of the newborn	1 (0.01)
Leiomyoma	1 (0.01)
Granuloma	1 (0.01)
Neurofibrosarcoma	1 (0.01)
Low-grade malignant myofibroblastoma	1 (0.01)
Total	6859 (100)

In order to classify these different types of NDPIGDs into more specific categories, we divided the lesion types into several groups according to their pathological characteristics. As shown in Table 2, the most frequently observed biopsied lesions were “hyperplastic lesions” (n = 2648), reaching up to 38.61% of all NDPIGDs. In “hyperplastic lesions”, the largest proportion was fibrous hyperplasia, followed by pyogenic granuloma. Due to the large number of OSCC cases, “malignant neoplasms” was second among all biopsied samples, with a percentage of 33.17%. The third and fourth most common categories were “oral potentially malignant disorders” (OPMD, n = 1214, 17.7%) and “autoimmune disorders” (n = 384, 5.6%). Among the OPMDs, those characterized by epithelial dysplasia and hyperplasia/hyperkeratosis were the most commonly encountered; of these “autoimmune disorders”, the largest number of biopsies were performed on pemphigoid. “Benign tumors” were also usually found in the gingiva, and papilloma seemed to be the most common. When it comes to “genetic lesions”, it included only one diagnosed type, namely hereditary gingival fibromatosis, with an occurrence of 0.58%. “Genetic lesions” was followed by “allergic reaction diseases” and “infectious diseases”, which included only four cases in total. There were still some diagnosed types that could not be allocated into the above eight categories, including melanotic macules, exogenous pigmentation, vascular malformations, hemangioma and granuloma.

**Table 2:** The number and frequency of different biopsied NDPIGDs according to pathological nature

Pathological nature	Histopathological diagnosis	Total n (%)
Hyperplasic lesions	Fibrous hyperplasia, Pyogenic granuloma, Verruciform xanthoma, Peripheral giant cell granuloma, Drug-induced gingival enlargements	2648 (38.61)
Malignant neoplasms	Oral squamous cell carcinoma, Malignant melanoma, Malignant lymphoma, Soft tissue sarcoma, Spindle cell sarcoma, Metastatic carcinoma, Malignant peripheral neurolemmoma, Leukemia, Myoepithelial carcinoma, Low-grade malignant myofibroblastoma, Neurofibrosarcoma	2275 (33.17)
Oral potentially malignant disorders	Epithelial dysplasia, Epithelia hyperplasia/ Hyperkeratosis, Lichen planus, Lichenoid lesion	1214 (17.7)
Autoimmune disorders	Pemphigoid, Pemphigus	384 (5.6)
Benign neoplasms	Papilloma, Peripheral ameloblastoma, Peripheral odontogenic fibroma, Nevus, Myofibroma, Neurofibromatosis, neurolemmoma, Inflammatory myofibroblastic tumor, Fibrous histiocytoma, Lipoma, Solitary fibrous tumor, Congenital epulis of the newborn, Leiomyoma	191 (2.78)
Genetic lesions	Hereditary gingival fibromatosis	40 (0.58)
Allergic reaction diseases	Plasma cell gingivitis	2 (0.03)
Infectious diseases	Tuberculosis	2 (0.03)
Other	Melanotic macules, Exogenous pigmentation, Vascular malformations, Hemangioma, Granuloma	103 (1.50)
Total		6859 (100)

**Fig. 1:**
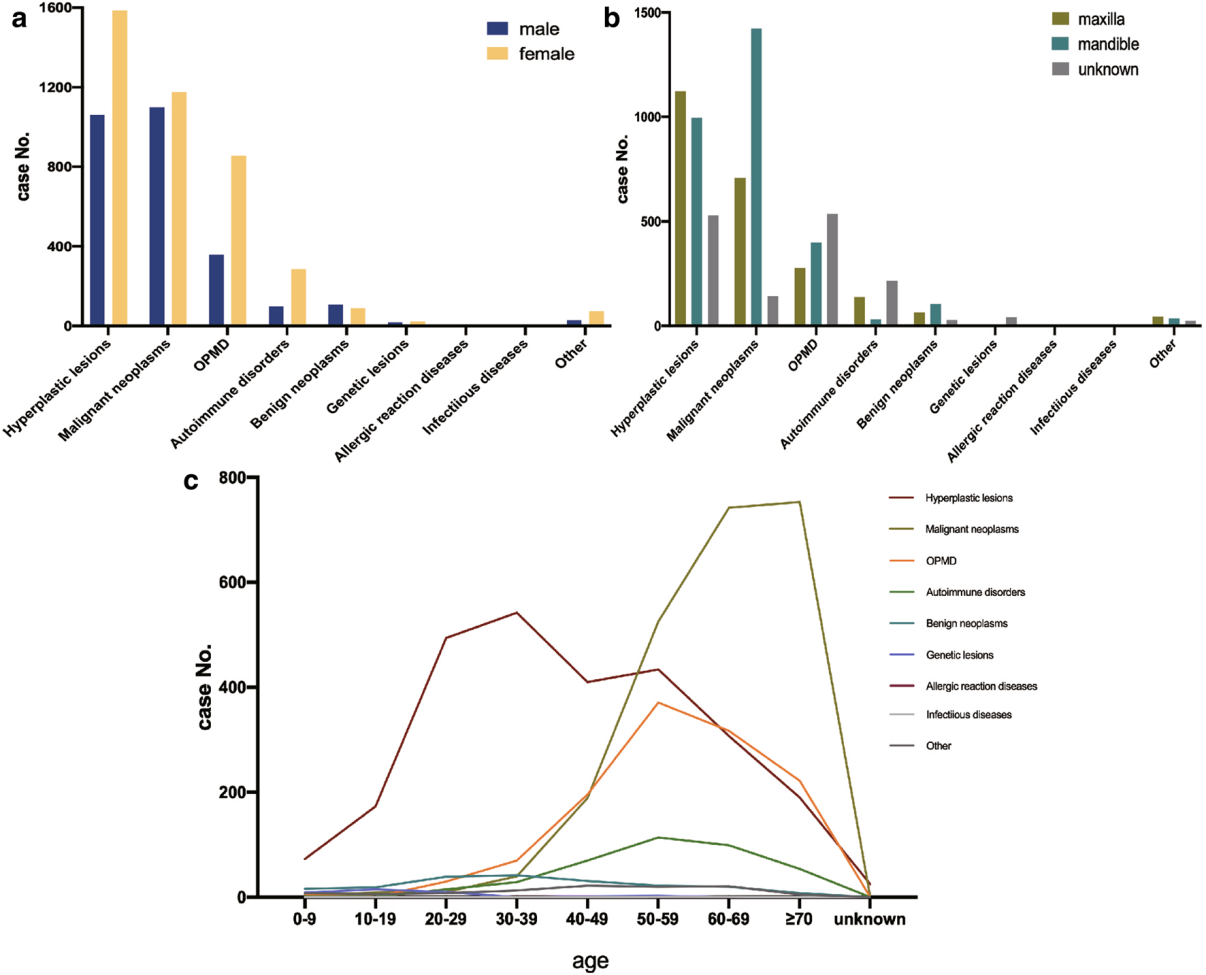
Demographic information of all patients with NDPIGDs based on pathological characteristics. **a** Bar chart showing gender distribution of biopsied NDPIGDs. **b** Bar chart showing distribution of location of biopsied NDPIGDs. **c** Point-fold line chart illustrating the age distribution of biopsied NDPIGDs

Next, we summarized the demographic information including gender, location, and age of all the categories based on the pathological nature. As shown in Fig. 1a, females showed a higher prevalence (59.64%) of NDPIGDs than males (40.36%). Gender ratios of different lesion groups were significantly different (*p* < 0.0001). All categories were more common in females, except benign neoplasms, which were more frequently observed in males. “Hyperplasia lesions” were the most common category for females and the second most frequent category for males. “Malignant neoplasms” followed and were found to have a high prevalence in females. “OPMDs” and “autoimmune disorders” both exhibited significant differences between genders, with a higher number of samples in females than in males. “Benign neoplasms”, in contrast, were more commonly observed in males. Of the 40 biopsied genetic lesion samples, 18 samples were those of males and 22 were of females. Due to the limited number of “allergic reaction diseases” category and “infectious diseases” category, gender differences were not statistically analyzed.

The distribution of location is also summarized. As shown in Fig. 1b, different categories of NDPIGDs showed preference for certain locations, and there was significant difference of location distribution among different groups (*p* < 0.0001). “Hyperplasic lesions” had a slightly higher rate in the maxilla (n = 1123) than in the mandible (n = 996). While “malignant neoplasms” cases were more likely to occur in mandible (n = 709), but were almost twice as many as than in the maxilla (n = 1423). Apart from a number of OPMD cases that had no location information reported, the remaining cases were more prevalent in the mandibular gingiva (n = 537), compared with the maxillary gingiva (n = 399). “Autoimmune disorders” were more frequent in the maxilla (n = 138), while “benign neoplasms” were located slightly more in the mandible (n = 106) compared with the maxilla (n = 63). Specifically, hereditary gingival fibromatosis affected the entire gingiva without any preference for a certain location. Therefore, we did not divide the biopsied samples into specific gingiva locations. “Allergic lesions and “infectious diseases” were also not analyzed due to the small number of samples.

As shown in Fig. 1c, biopsied NDPIGDs were also diagnosed within a wide age range, from 0 to 86 years old. Age ratios of different lesion groups were significantly different (*p* < 0.0001). “Hyperplasia lesions” were observed in all age groups, with the highest prevalence in the age range of 20–59 years. “Malignant neoplasms” showed a prevailing concentration in elderly patients of 50–69 years old. A similar conclusion could also be drawn in the “OPMD” category, showing that the majority of these lesions were diagnosed in 30–69-year-old patients and exhibited a peak incidence among the patients in the age group of 50–59 years old. “Autoimmune disorders” were not frequently diagnosed in patients under the age of 40, and patients in the age group of 40–70 years were the most prevalent. “Benign neoplasms” were not specifically diagnosed in any specific age group. In contrast to the categories mentioned above, “genetic lesions” were mostly diagnosed under the age of 29 years. “Allergic reaction diseases”, “infectious diseases” and other non-divided samples were scattered among all age groups.

We finally classified all NDPIGDs into groups according to the new classification of gingival health and gingival diseases/conditions approved in 2018. As shown in Table 3, hereditary gingival fibromatosis was classified into the “genetic/developmental disorders” category; tuberculosis was considered a “specific infections”; pemphigoid, lichen planus, pemphigus, plasma cell gingivitis and granuloma were categorized into “inflammatory and immune conditions”; the “reactive processes” category included fibrous hyperplasia, pyogenic granuloma and peripheral giant cell granuloma; the predominant category was “neoplasms”, which included OSCC, epithelial dysplasia, epithelia hyperplasia/hyperkeratosis and malignant lymphoma; “gingival pigmentation” included melanotic macules and exogenous pigmentation. However, there were numerous types that could not be classified according to the new system and were grouped into “not otherwise specified”. This category included malignant melanoma, verruciform xanthoma, papilloma, peripheral ameloblastoma, and another 23 diagnoses, most of which were malignant/benign neoplasms with a frequency of 7.07%.

**Table 3:** The number and frequency of different biopsied NDPIGDs according to new classification scheme for periodontal and peri-implant diseases and conditions

New classification scheme for periodontal and peri-implant diseases and conditions	Histopathological diagnosis	Total n (%)
Genetic/ developmental disorders	Hereditary gingival fibromatosis	40 (0.58)
Specific infection	Tuberculosis	2 (0.03)
Inflammatory and immune conditions	Pemphigoid, Lichen planus, Pemphigus, Plasma cell gingivitis, Granuloma	677 (9.87)
Reactive processes	Fibrous hyperplasia, Pyogenic granuloma, Peripheral giant cell granuloma	2536 (36.94)
Neoplasms	Oral squamous cell carcinoma, Epithelial dysplasia, Epithelia hyperplasia/ Hyperkeratosis, Malignant lymphoma	3040 (44.32)
Gingival pigmentation	Melanotic macules, Exogenous pigmentation	79 (1.15)
Not otherwise specified	Malignant melanoma, Verruciform xanthoma, Papilloma, Peripheral ameloblastoma, Drug-induced gingival enlargements, Peripheral odontogenic fibroma, Soft tissue sarcoma, Spindle cell sarcoma, Nevus, Vascular malformations, Lichenoid lesion, Metastatic carcinoma, Myofibroma, Hemangioma, Malignant peripheral neurolemmoma, Neurofibromatosis, Neurolemmoma, Inflammatory myofibroblastic tumor, Leukemia, Myoepithelial carcinoma, Fibrous histiocytoma, Lipoma, Solitary fibrous tumor, Congenital epulis of the newborn, Leiomyoma, Neurofibrosarcoma, Low-grade malignant myofibroblastoma	485 (7.07)
Total		6859 (100)

## Discussion

Even though the majority of gingival diseases are caused by bacterial biofilms, it should also be borne in mind that numerous types of lesions with different etiological factors could also affect the gingival health, or even become life-threatening [12]. These lesions were not initially induced by dental pathogens; however, the outcomes of these lesions are also largely influenced by bacterial plaque. In this study, we carried out a single-institute retrospective study to analyze the frequency and distribution of NDPIGDs in a Chinese population over the past 20 years. We adjusted and compared the current results to the new classification of gingival health and gingival diseases/conditions established in 2018.

The biopsied NDPIGDs in this study consisted of 43 types according to their pathological diagnosis. We then categorized them into nine groups based on their pathological characteristics. From the most common lesions to the least common were hyperplastic lesion, malignant lesions, OPMDs, autoimmune disorders, benign neoplasms, genetic lesions, allergic reaction diseases, infectious diseases, and other diseases that could not be categorized into the former groups.

Previous studies regarding the classification of biopsied gingival lesions mainly divided them into three categories: non-neoplastic lesions, benign lesions, and malignant lesions [2, 7, 8], and the majority of biopsied samples were non-neoplastic lesions. Consistent with their reports, our statistical results also showed that the largest number of NDPIGDs were hyperplastic lesions, with 38.61% of all lesions. Among them, the most common diagnosis type was fibrous hyperplasia, which accounted for nearly 30% of all lesions. As important types of the epulis, pyogenic granuloma and peripheral giant cell granuloma also showed relatively high frequencies. In particular, females showed a higher prevalence of hyperplastic lesions compared with males, which were less frequently occurred in the mandible than in the maxilla. In our results, hyperplastic lesions widely ranged between and 20–59 years of age, with an incidence peak between and 30–39 years; however, this is not in complete agreement with previous studies. In a similar study, the highest frequency appeared in patients between and 60–69 years [2], while two other studies pointed out that the majority of biopsied samples were between 30–39 years and 20–29 years [6, 18]. We assumed that the inconsistency was possibly caused by different criteria as well as pathological diagnosis habits. Furthermore, one of these studies merged reactive hyperplastic lesions and other inflammatory diseases into one group.

The largest inconsistency of the biopsied NDPIGDs were the “malignant neoplasms” category. In past research, the proportion of malignancies on the gingiva was reported to be 1–8% [2, 6, 11]. However, malignant neoplastic lesions represented up to 33.14% of all gingival lesions in our study. This high incidence was significantly different from that in earlier reports. Among them, Layfield et al. [6] represented the most comprehensive biopsied gingival lesions research that collected a total of 30,056 samples over a period of 24 years. In their study, the frequency of malignant neoplasms was as low as 1.4%. Such a huge difference demonstrated the difference in prevalence between Easterners and Westerners. Notably, when considering the distribution of the malignancies, the most prevalent neoplasm reported in our research and previous studies was the same, OSCC [19, 20], which accounted for 30.53% of all samples.

The former category was then followed by “OPMDs”, “autoimmune disorders”, and “benign neoplasms”. The category “OPMD” was defined as a significant group of mucosal disorders that may precede the diagnosis of OSCC [21], which included oral leukoplakia, oral erythroplakia, oral lichen planus, and newly added oral lichenoid lesions. Depending on the presence or absence of epithelial dysplasia, oral leukoplakia was divided into epithelial hyperplasia/hyperkeratosis and epithelial dysplasia, with the frequencies of 6.36% and 6.95%, respectively. In this study, lichen planus and oral lichenoid lesions were placed under the OPMD category. In total, the total number of OPMDs reached 1214, accounting for 17.7% of all NDPIGD cases. This ratio was remarkably higher than those previously reported [11, 22].

The “autoimmune disorders” category was put forward for the first time in our work and pemphigoid and pemphigus were categorized under the group. In former studies, pemphigoid and pemphigus were not specifically mentioned, either due to the low frequency or they were classified into “mucocutaneous disorders” [11]. However, the two lesions in our work account for 5.6% of all samples, and we believed “mucocutaneous disorders” was ambiguous for describing the type, as many other diseases like oral leukoplakia might be confused. Benign neoplasms, on the other hand, are not commonly seen in the gingiva. Of all 6859 biopsied samples, only 197 cases of different diagnosis types were observed. While malignant neoplasms reached a peak incidence in those aged over 60 years, benign tumors were distributed separately.

Hereditary gingival fibromatosis is a frequently common genetic disease seen in the periodontal clinic. As the present research is mainly concentrated on biopsied cases, the frequency (0.58%) might be largely underestimated compared to the actual incidence in clinical settings. Hernández-Ríos’s team [11] classified papilloma into “infectious lesions” while we placed papilloma into benign neoplasms and tuberculosis was the only diagnosed type in the “infectious lesions” group. Plasma cell gingivitis was also found in two female patients.

Etiologies of gingival diseases were first introduced into the classification of periodontal diseases and conditions in 1999 [3]. This classification added gingival disease for the first time and divided them into two subcategories based on the initial factor, that is, dental plaque-induced gingival diseases and non-plaque-induced gingival diseases. Hernández-Ríos et al. [11] distributed the biopsied non-plaque-induced gingival lesions based on the 1999 classification of periodontal diseases and conditions. However, as they presented, over 90% of biopsied gingival lesions could not be categorized according to this classification, including the most prevalent groups (hyperplastic lesions and malignant neoplasms). To a large extent, these results reflect the shortcomings of this classification system.

To the best of our knowledge, the present study is the first report on the frequency and distribution of biopsied NDPIGDs in a Chinese population. This is also the first study to compare the pathological nature classification and the latest 2018 classification of gingival diseases. The epidemiology of the pathological diagnosis of NDPIGDs shared numerous similarities as well as certain differences with previous reports. Specifically, when categorizing pathologically diagnosed biopsied lesions with the pathological nature classification and the new classification of gingival diseases, there were some discrepancies. Lichen planus and lichenoid lesions were grouped under “OPMD”, however, lichen planus was grouped into “inflammatory and immune conditions” according to the new classification of gingival diseases, and lichenoid lesions were not specifically categorized. These results demonstrated the inconsistencies between the two classification systems, which may cloud clinicians’ judgments. It should be noted that the classification system may be helpful for clinicians in early diagnosis and subsequent treatments. A classification system with more specific categories that based on prevalence, specificity and mortality is highly recommended.

However, there are still some limitations for this study that require further investigation. First, our samples were all from the same hospital and the study is a single-center retrospective research. Second, we lack complete clinical and demographic information of many patients. In addition, our study only investigated the biopsied samples with definite pathological diagnosis; those gingival lesions that can be recognized through clinical performance without biopsy were not included into the research. Overall, the present research represented the first report of the distribution and frequency of biopsied NDPIGDs in a Chinese population as well as the first report to compare the prevalence using the 2018 classification of gingival diseases.

## Conclusions

In conclusion, our retrospective study demonstrated that hyperplastic lesions and malignant neoplasms were the most common biopsied NDPIGDs in a Chinese population based on a single-institute analysis. Certain differences did exist among different classification systems, and future classification schemes could be improved by taking more comprehensive information (i.e., prevalence, mortality, and specificity) into consideration.

## Data Availability

The datasets used and/or analyzed during the current study are not publicly available due to the absence of a public storage online platform in our hospital, but are available from the corresponding author on reasonable request.
